# Sputum completion and conversion rates after intensive phase of tuberculosis treatment: an assessment of the Rwandan control program

**DOI:** 10.1186/1756-0500-5-357

**Published:** 2012-07-16

**Authors:** Felix R Kayigamba, Mirjam I Bakker, Veronicah Mugisha, Michel Gasana, Maarten F Schim van der Loeff

**Affiliations:** 1 INTERACT, CPCD, PO Box 2181, Kigali, Rwanda; 2 Royal Tropical Institute, KIT Biomedical Research, Mauritskade 63, 1092 AD, Amsterdam, Netherlands; 3 ICAP, Mailman School of Public Health, Columbia University, P.O. Box 3989, Kigali, Rwanda; 4 Rwanda National TB Control Program (PNILT), Ministry of Health, P.O. Box 84, Kigali, Rwanda; 5 Center for poverty-related communicable diseases (CPCD) and Center for Infection and Immunity Amsterdam (CINIMA), AMC, P.O. Box 22700, 1100 DE, Amsterdam, Netherlands; 6 Public Health Service of Amsterdam (GGD), P.O. Box 2200, 1000 CE, Amsterdam, Netherlands

**Keywords:** Sputum smear examination, Mycobacterium tuberculosis, Pulmonary tuberculosis, Africa

## Abstract

**Background:**

In Rwanda tuberculosis (TB) is one of the major health problems. To contribute to an improved performance of the Rwandan National TB Control Program, we conducted a study with the following objectives: (1) to assess the completion rate of sputum smear examinations at the end of the intensive phase of TB treatment; (2) to assess the sputum conversion rate (SCR); (3) to assess associations between smear completion rate or SCR with key health facility characteristics.

**Methods:**

TB registers in 89 health facilities in five provinces were reviewed. Data of new and retreatment smear-positive pulmonary TB (PTB+) cases registered between January and June 2006 were included in the study. Data on key characteristics of the selected health facilities were also collected.

**Results:**

Among 1509 new PTB + cases, 32 (2.1%) had died by 2 months, and 178 (11.8%) had been transferred-out. Among the remaining 1299 patients, a smear examination at month 2 was done in 1039 (smear completion rate 80.0%). Among these 1039, 852 (82.0%) had become smear-negative. The smear completion rate and SCR varied considerably between health facilities. A high number of new PTB cases at a health facility was the only significant predictor of a low completion rate, while the only independent factor associated with low sputum conversion rates was rural (vs. urban) location of the health facility.

**Conclusions:**

In Rwanda, too few patients get a smear examination after 2 months of TB treatment; the SCR among those with smear results was adequate at 82%. A high number of new TB patients at a health facility was a significant predictor of a low completion rate. The national TB control program should design strategies to improve completion rates.

## Background

A third of the world’s population is infected by *Mycobacterium tuberculosis* and it is estimated by the World Health Organization (WHO) that 1.45 million people die each year from tuberculosis (TB) [1]. Ninety-five percent of TB cases and 98% of TB deaths occur in developing counties [2]. In Rwanda TB is a major health problem: in 2010 the TB notification rate was 72 per 100,000 population [3].

The sputum conversion rate (SCR) is the percentage of smear-positive pulmonary TB (PTB+) cases registered in a specified period that converted to smear negative status after the standard two months of the intensive phase of treatment. WHO recommends its use as a useful indicator for TB control programs in monitoring the TB program performance, and as a trigger for rigorous assessment in patients with still positive smears [4]. Even in well functioning national TB programs 25% of initially PTB + patients may still be smear-positive at the end of the intensive phase of treatment, despite good adherence and supervised medication [5].

Once the sputum smear has become negative, patients are considered to be no longer infectious [6]. Those failing to achieve sputum conversion after 2 months are more likely to have poor treatment outcomes [7-10]. If the sputum smear is still positive after 2 months of treatment, the intensive phase is extended for one more month [5,11].

Studies to evaluate the SCRs and the predictive factors for failure to convert have been conducted in several countries in sub-Saharan Africa and elsewhere, but not in Rwanda [7-10,12-23].

The objectives of this study were to assess the proportion of patients in Rwandan health facilities for whom a smear examination after the intensive phase of treatment was done (completion rate), to assess the sputum smear conversion rate among new cases and retreatment PTB + cases, and to assess whether key health facility characteristics are associated with smear completion rate or with SCR in the Rwandan program.

## Methods

### Procedures

We conducted a revi*ew* of TB treatment registers in health facilities. The diagnosis of PTB in Rwanda is mainly by sputum microscopy. The Kinyoun staining technique was used during the study period (in 2009 Ziehl-Neelsen staining was adopted). All patients attending out-patient departments with cough for three weeks or longer, were considered TB suspects and requested to submit three sputum samples for microscopic examination (usually on two consecutive days). At least two positive smears were required to confirm a suspected TB case as PTB + [24] (in 2009 guidelines were changed to two weeks of cough and at least one positive smear) [11].

### Treatment Regimen

In Rwanda, TB treatment is directly supervised. The treatment regimen for new PTB + cases in Rwanda consists of a two month intensive phase with a daily dose of fixed-dose combination tablets containing Rifampicin (R), Isoniazid (H), Pyrazinamide (Z) and Ethambutol (E), followed by a four months continuation phase of daily RH. To assess sputum conversion, sputum microscopy is done after the second (C2), fourth and sixth month of treatment. PTB + cases are expected to convert to negative sputum status after two months of intensive TB treatment.

TB retreatment cases include cases that relapsed after, defaulted during or failed on first line treatment. These are treated with an eight-month regimen consisting of daily streptomycin (S) and RHZE for two months, daily RHZE for one month, and daily RHE for five months. Sputum microscopy to assess conversion is done after 3 months of intensive treatment [11].

### Study setting

To achieve representativeness for Rwanda, this study was performed in three administrative districts selected from each of the five provinces. Kigali city has three districts while the other four provinces have more. All three Kigali city districts were included in the study while the three districts with the highest numbers of patients registered during the period we studied were selected from each of the other four provinces. All TB diagnostic health facilities (n = 89) in the selected 15 districts were included in the study.

### Data collection and management

At each selected health facility aggregated data of TB patients registered during the study period (January-June 2006) were abstracted. Data included the numbers of patients whose sputum was not evaluated, who converted to negative sputum status, who did not convert, who died, who were lost to follow-up or were transferred out; data for new PTB + cases and smear positive retreatment cases were recorded separately. The PTB patients in this study form around 70% of new smear-positive PTB cases and about 75% of re-smear-positive PTB treatment cases diagnosed nation-wide in the study period.

Additionally data on health facility characteristics were collected through structured interviews with the heads of the health facilities: the type (hospital/health centre), the location (urban/rural), the ownership status (private, mission, government), the highest level of health care workers in the TB department, and the date of the most recent on-the-job training for staff in the TB department. The educational levels of health care workers included university graduate (grade A0), registered nurse (grade A1), ordinary nurse (grade A2), and auxiliary nurse (grade A3). Refresher training courses on TB clinical care were considered as on-the-job-training.

Data entry was done with EpiInfo version 3.3 (CDC, Atlanta, USA) and analysis with Stata 11 (StataCorp, College Station, Texas, USA).

### Statistical Analysis

The coverage of sputum microscopy for PTB + cases at the end of the 2-month intensive phase (smear completion rate) was calculated as follows. The denominator was the number of new PTB + cases initially registered minus those who had died or had been transferred out during the first two months of treatment. The numerator was the number of new PTB + cases in whom a sputum examination at C2 was done [10]. The SCR was calculated as the proportion of patients who had become smear-negative out of all patients of whom a 2-month sputum smear was available [8-10,15,21,25]. Thus, the SCR can vary between 0 and 1. Smear completion rate and SCR were calculated similarly for retreatment cases, except that the period between start of treatment and expected smear examination was three rather than two months.

Because the distributions of smear completion rates and SCRs were non-normal and logit transformation could not redress this, linear regression with smear completion and SCR as outcomes was not done. Instead categorical variables were created based on cut-offs of completion rate and SCR. A completion rate of less than 90% in a health facility was regarded as a poor completion rate. An SCR of less than 75% was regarded as a low SCR [5]. Logistic regression analyses were done to assess the associations between health facility characteristics and low smear completion rate and low SCR. The natural logarithm of the total number of new TB patients in each health facility was used as a weight in the regression analyses, giving more weight to health facilities with more patients. Odds ratios were derived with 95% confidence intervals and P values of <0.05 were considered statistically significant.

### Ethics

The Rwanda National TB Control Program (PNILT) approved this study. All heads of selected health facilities agreed to participate. Informed consent of patients was not sought, as we collected and analysed anonymised aggregated routine program data.

## Results

### New cases

The total number of registered new PTB + cases between January and June 2006 in the 89 health facilities was 1509. Overall 1039 (69%) of the 1509 new PTB + cases had sputum microscopy done after two months of intensive phase treatment (Table 1). As smear results were not expected from patients who had died (n = 32) or who had transferred out to another clinic (n = 178), the completion rate was 80.0% (1039/1299); this varied between 17.6% and 100% per health facility (median 90.6%). Thirty-three sites had 100% coverage.

**Table 1:** Sputum conversion rate against sputum completion rate among new TB cases for 89 health facilities

	**New patients**^**a**^	**Retreatment patients**^**b**^
Total number of patients	1509	206
No sputum examination expected after intensive phase	210	30
Died < 2 months	32 (15.2%)	5 (16.7%)
Transferred out < 2 months	178 (84.8%)	25 (83.3%)
Sputum examination expected after intensive phase	1299	176
Sputum smear done	1039 (80.0%)	133 (75.6%)
Sputum smear not done	260 (20.0%)	43 (24.4%)
Sputum smear done after intensive phase	1039	133
Sputum smear negative	852 (82.0%)	109 (82.0%)
Sputum smear positive	187 (18.0%)	24 (18.0%)

^a^ Duration of intensive phase is 2 months.

^b^ Duration of intensive phase is 3 months.

Sputum conversion was observed in 82.0% (852/1039) of the patients. The SCR among new PTB + cases varied per health centre between 0 and 100% with a median of 84.0% (IQR 67.9-95.9%). At 21 sites all examined cases had converted.

**Figure 1:**
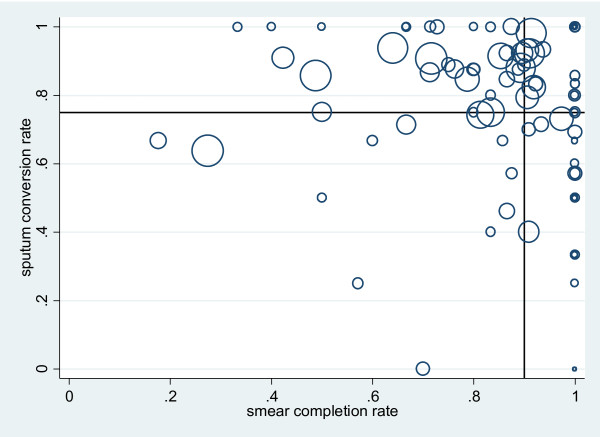
**Sputum conversion rate against sputum completion rate among new TB cases for 89 health facilities.** The size of the circles is proportional to the number of new TB cases diagnosed at each of the 89 health facilities in Rwanda. The horizontal line at 0.75 indicates the cut-off for an adequate sputum conversion rate. The vertical line at 0.9 indicates the cut-off for an adequate smear completion rate

Figure 1 shows the variation of completion rate and SCR in all health facilities. Health facilities that performed well on one indicator did not necessarily perform well on the other indicator.

### Retreatment cases

The total number of smear-positive retreatment patients was 206. After the three months intensive phase, a sputum smear was available for 133 out of the 176 in whom it should have been done (completion rate 75.6%). Smear conversion was observed in 109 (82.0%); 24 had not converted (Table 1).

### Health facilities

Out of the 89 health facilities included in the study, 70 (78.7%) were health centres and 19 (21.3%) were hospitals. The number of newly registered PTB + cases per health facility in the six-month period varied between 0 and 71 (median 8). One site did not have any new cases and was excluded from further analyses. The number of registered retreatment cases varied between 0 and 29 (median 1). Forty-one sites did not register any retreatment cases in the study period.

### Analysis of associations between health facility attributes and completion rate among new cases

Forty-three (48.9%) health facilities had completion rates below 90%. In bivariable analysis, a completion rate below 90% was associated with hospital (versus health centre) (p = 0.025), urban location of facility (p = 0.008), the province (p = 0.045), and the number of new patients diagnosed at the centre (p = 0.001) (Table 2). In multivariable logistic regression, the only significant predictor of a low completion rate was a high number (≥15) of new PTB cases at the health facility.

**Table 2:** Associations between poor sputum completion rates among new sputum-smear positive PTB patients and health facility characteristics at 88 health facilities, Rwanda, 2006

	**Number (%)**	**Number (%) with smear completion rate <90%**	**Bivariable Odds Ratio**^**a**^	**95%CI**	**P**^**b**^	**Multi-variable Odds Ratio**^**a**^	**95%CI**	**P**^**b**^
Overall	88 (100%)	43 (48.9%)						
Category of health facility					0.025			
Health centre	69 (78.4%)	30 (43.5%)	1					
Hospital	19 (21.6%)	13 (68.4%)	2.0	1.1-3.7				
Location of health facility					0.008			
Rural	60 (68.2%)	25 (41.7%)	1					
Urban	28 (31.8%)	18 (64.3%)	2.1	1.2-3.8				
Health facility status					0.311			
Mission/private	40 (45.5%)	19 (47.5%)	1					
Public	48 (54.5%)	24 (50.0%)	1.3	0.8-2.3				
Most recent on-the-job training for TB staff								
Before 2006 or none at all	47 (53.4%)	22 (46.8%)	1		0.643			
2006	41 (46.6%)	21 (51.2%)	1.1	0.7-2.0				
Location of facilities (Province)					0.045			
East	13 (14.8%)	8 (61.5%)	1					
Kigali	17 (19.3%)	11 (64.7%)	1.4	0.6-3.6				
North	18 (20.5%)	4 (22.2%)	0.4	0.1-1.0				
West	22 (25.0%)	11 (50.0%)	0.9	0.3-2.2				
South	18 (20.5%)	9 (50.0%)	1.0	0.4-2.5				
Number of new PTB cases in 6-month period					0.001			0.001
1-5	32 (36.4%)	9 (28.1%)	1			1		
6-14	27 (30.7%)	14 (51.9%)	2.0	0.9-4.4		2.0	0.9-4.4	
≥15	29 (33.0%)	20 (69.0%)	4.1	1.9-8.8		4.1	1.9-8.8	

^a.^ The natural log of the number of newly registered PTB cases was used as weight in the regression analysis; ^b^ Based on the log likelihood ratio. One site did not register any new cases during the study period and was not included in the results in this table. *PTB* pulmonary tuberculosis; *TB* tuberculosis; *CI* confidence interval; *HCW* health care worker.

### Analysis of associations between health facility attributes and SCR among new cases

Twenty-eight (31.8%) of health facilities had smear conversion rates below 75%. In bivariable analysis hospitals (versus health centres) and urban health facilities (versus rural health facilities) were less likely to have low SCR (p = 0.017 and p = 0.001, respectively; Table 3). In multivariable logistic regression the only independent factor associated with a low SCR was rural location of the health facility (p = 0.001).

**Table 3:** Associations between poor sputum smear conversion rates among new sputum-smear positive PTB patients and health facility characteristics in 88 health facilities, Rwanda, 2006

	**Number (%)**	**Number (%) with smear conversion <75%**	**Bivariable Odds Ratio**^**a**^	**95%CI**	**P**^**b**^	**Multi-variable Odds Ratio**^**a**^	**95%CI**	**P**^**b**^
Overall	88 (100%)	28 (31.8%)						
Category of health facility					0.017			
Health centre	69 (78.4%)	24 (34.8%)	1					
Hospital	19 (21.6%)	4 (21.1%)	0.4	0.2-0.9				
Location of health facility					0.001			0.001
Rural	60 (68.2%)	23 (38.3%)	1			1		
Urban	28 (31.8%)	5 (17.9%)	0.3	0.2-0.6		0.3	0.2-0.6	
Health facility status					0.216			
Mission/private	40 (45.5%)	13 (32.5%)	1					
Public	48 (54.5%)	15 (31.3%)	0.7	0.4-1.2				
Most recent on-the-job training for TB staff								
Before 2006 or none at all	47 (53.4%)	15 (31.9%)	1		0.235			
2006	41 (46.6%)	13 (31.7%)	1.4	0.8-2.6				
Location of facilities (Province)					0.729			
East	13 (14.8%)	4 (30.8%)	1					
Kigali	17 (19.3%)	4 (23.5%)	0.6	0.2-1.7				
North	18 (20.5%)	8 (44.4%)	1.0	0.4-2.9				
West	22 (25.0%)	7 (31.8%)	1.1	0.4-2.9				
South	18 (20.5%)	5 (27.8%)	0.9	0.3-2.4				
Number of new PTB cases in 6-month period					0.499			
1-5	32 (36.4%)	11 (34.4%)	1					
6-14	27 (30.7%)	9 (33.3%)	1	0.4-2.2				
≥15	29 (33.0%)	8 (27.6%)	0.7	0.3-1.5				

The data in this table are based on new sputum smear positive patients of whom a control sputum smear was available after 2 months of intensive treatment. One site did not register any new cases during the study period and was not included in the results in this table. ^a^ The natural log of the number of newly registered PTB cases was used as weight in the regression analysis. ^b^ Based on the log likelihood ratio. *PTB* pulmonary tuberculosis; *TB* tuberculosis; *CI* confidence interval; *HCW* health care worker.

## Discussion

The SCR among new PTB + cases after the intensive phase of treatment was 82% among both new and retreatment cases. The WHO standard for a well functioning national TB program is a conversion rate of at least 75% among new PTB+ [5], so the Rwandan program appears to be performing well in this respect.

Comparisons of SCR between studies are challenging because different researchers have used different definitions of SCR. Some include all newly diagnosed smear-positive PTB patients in the denominator [18-20], while others only include those of whom a smear result was available after the intensive phase of treatment [7-9,15,21,25]. The first approach can yield a relatively low SCR if the program is not successful in performing smears after the intensive phase; in those cases the SCR becomes hard to interpret. Therefore we opted to calculate both a smear completion rate (reflecting the ability of the program to get sputum smears done) and the SCR (reflecting the ability of treatment to sterilise the sputum).

The SCR among new PTB patients in our study is lower than those found in studies performed in Tanzania (98.6%) [21], China (95.0%) [8], Cameroon (86.6%) [9], but higher than in a refugee camp in Thailand (75.0%) [7] and Taiwan (80.0%) [25]. The SCR is similar to that observed in Burkina Faso (92.1%) [10] and Uganda (76.0%) [15].

A number of studies examined patient level factors in relation to sputum conversion. Important factors were shown to be higher pre-treatment sputum grade [9,15-17,19,22,23] and extensive disease involvement on chest X-Ray [19,20]. Some studies found that older age [9,16,19] and male sex [10,18,21] were related to failure to convert. The effect of initial drug resistance on sputum conversion differed between studies [13,15,16,22,23] Co-infection with HIV has not been reported to be a significant factor for SCR or time to conversion [14-16,21,23]. So, individual patient level factors are important determinants of SCR. However in our study, we examined whether health facility factors were associated with SCR, using aggregate data. Sputum conversion was significantly associated with the location of the health facility at which patients were treated: urban sites more often had adequate SCRs than rural sites. Underlying factors could be proximity and lower travel cost [26-28]. Lower socioeconomic status [27-29], illiteracy or insufficient knowledge about TB disease [28-30] are more frequent in rural areas. Although quality control for smear microscopy is done by the National Reference Laboratory, it is possible that the variation in SCR between centres is caused by varying quality of the reading of smears.

Failure to become smear-negative might be due to (multi-) drug-resistant TB [22]. In Rwanda a national representative study conducted in 2004–5 estimated a prevalence of 3.9% of MDR among new smear-positive PTB patients [31]. This is a relatively high prevalence, but too low to significantly impact on the SCR in Rwanda.

The completion rate of sputum smear examinations after the intensive phase of treatment was 80%, which is low. Health facilities with a high number of new TB patients were more likely to have a low completion rate; this can be attributed to heavy workload in facilities that are under-staffed. District hospitals in Rwanda serve as referral facilities for all satellite diagnostic and treatment centres in their respective catchment zones. In addition to receiving many patients in routine care, these hospitals receive complicated clinical cases from health centres, including TB cases; this makes the workload heavier. Although our study did not find significant associations between level of education of health staff and completion rates, upgrading the level of education for existing staff or additional recruitments of some A1 staff at health facilities may improve smear completion rates and other standards of care. The majority of health facility staff did not get regular technical supervision or regular on-the-job training for TB management. Results may improve through regular on-the-job training for health care workers. Health facilities that showed a high smear completion rate did not necessarily have a high conversion rate and vice versa; very few clinics had low rates on both measures. These measures are influenced by different health care factors: the smear completion rate can be influenced by quality of the work of the clinical staff, and the conversion rate by the quality of the laboratory work.

This study has some limitations. The data were collected in 2006 and possibly smear completion rate has improved since then. The sample size of this study was limited, although this was a representative sample of all health facilities diagnosing and treating TB, the power to detect significant associations was limited. Error in completing records may not be uncommon in routine clinics and may have led to bias in data collection. Individual patient factors like age, sex, smear grade and adherence are –or may be- related to smear conversion; this study focused on health sector factors, and thus omitted knowingly important factors from the analysis.

## Conclusions

In Rwanda the coverage of smear sputum examination after the end of the intensive phase of TB treatment is too low and efforts are needed to increase this. The SCR in Rwanda at 82% is adequate. A high number of new TB patients at a health facility was a significant predictor of a low completion rate. The national TB control program should design strategies to improve completion rates, with a focus on the busiest clinics.

## Competing interests

The authors declare that they have no competing interests.

## Authors’ contributions

VM, FK and MG designed the study. FK carried out data collection. FK, MB, and MSvdL conducted the statistical analysis. FK wrote the first draft of the paper. All authors contributed to revisions and saw and approved the final version of the manuscript.
